# Outcomes of a cemented modular rotational-hinge design as the final implant in a two-stage replacement due to chronic knee periprosthetic joint infection

**DOI:** 10.1007/s00402-024-05516-x

**Published:** 2024-09-17

**Authors:** Marta Pérez, Matías Vicente, Carles Amat, Berta Lahoz, Lluís Carrera, Pablo S. Corona

**Affiliations:** 1 Universitat Autònoma de Barcelona (UAB), Orthopaedic Surgery Department, Vall d’Hebron University Hospital, Pg. Vall d’Hebron 119-129, 08035 Barcelona, Spain; 2 Universitat Autònoma de Barcelona (UAB), Bellaterra, Barcelona, Spain; 3 Septic and Reconstructive Surgery Unit (UCSO), Orthopaedic Surgery Department, Vall d’Hebron University Hospital, Barcelona, Spain; 4 Musculoskeletal Tissue Engineering Group, Vall d’Hebron Research Institute, Barcelona, Spain

**Keywords:** Periprosthetic joint infection, Knee arthroplasty, Exchange arthroplasty, Two-stage exchange arthroplasty, Hinge knee arthroplasty, Incisional closed negative pressure wound therapy

## Introduction

Total Knee Arthroplasty (TKA) is considered a highly successful surgical intervention for osteoarthritic knee pain. It is not, however, free of complications, with data suggesting Periprosthetic Joint Infection (PJI) as a complication on the rise.

Although direct exchange arthroplasty has been proven a reliable strategy in certain scenarios, two-stage reconstruction has been the widely accepted model of care, with reported success rates exceeding 90% in early publications [1, 2]. However, a superior two-stage treatment algorithm is lacking, and management of chronic knee PJI remains controversial. Of greater concern is the fact that the true outcome of this strategy is not as successful as previously published, with series showing an infection control rate in the neighbourhood of 75% or below [3]. This reality creates an obligation to explore new pathways to manage this difficult-to-treat scenario.

Unrelated to the adopted exchange strategy, the quality of the surgical debridement is of essential importance in achieving infection control. Unfortunately, aggressive debridement involves possible impairment of collateral ligament integrity and function, affecting the choice of revision implant to be used during the reconstructive stage. In such an unfavourable scenario, rotational-hinge knee designs have produced favourable outcomes [4, 5] but reports on results with this implant design in treatment of knee PJI are scarce in the scientific literature [6].

Based on our experience in managing patients with complex knee infections, we have evolved a comprehensive two-stage protocol which involves the use of a single model of cemented modular rotational hinge (CMRH) prosthesis as the final implant.

Considering all factors discussed above, we sought to analyse outcomes when using a CMRH revision arthroplasty in treatment of chronic knee PJI, focusing on (1) infection control rate after a two-stage procedure, (2) mid-term implant survivorship, and (3) complications related to its use in a septic context. Additionally, a univariate analysis was carried out to identify potential risk factors associated with failure of the proposed treatment protocol.

## Materials and methods

### Study design

After Institutional Review Board (IRB) approval, we reviewed our centre’s database and identified all consecutive patients who had been diagnosed with chronic knee PJI and who had undergone a two-stage exchange arthroplasty with implantation of a specific model of CMRH prosthesis (Endo-Model®-M, Waldemar Link GmbH & Co.®; Hamburg; Germany) as the final implant, from January 2010 through December 2021.

We included all patients operated upon by any of the four surgeons in our dedicated septic unit, provided that the patients met the following criteria: (1) Established diagnosis of chronic PJI according to an internationally accepted definition [7]; (2) Two-stage exchange arthroplasty strategy; (3) Use of an Endo-Model®-M cemented implant during the second stage; (4) Minimum follow-up of 24 months after the second stage. Patients with no post-surgery tracking data, monoblock rotational hinge implants, uncemented implants, cases of distal femur substitution managed with the same implant design and cases who did not fit all inclusion criteria were excluded.

### Variables

The primary end-points were infection control rate and Endo-Model®-M survivorship. Patient demographic variables, American Society of Anaesthesiologists (ASA) Scale, Charlson’s Comorbidity Index (CCI) and McPherson’s host classification were retrospectively collected. First-stage- and second-stage-related variables such as type of spacer, final modular reconstruction and isolated microorganisms were reviewed. Complications and reoperations during the spacer stage were collected as well as post-second-stage data: complications, need for unexpected reinterventions, infection relapse and prosthesis loosening.

#### Definitions

1. *Infection:* Chronic PJI cases were diagnosed according to the 2021 EBJIS criteria definition [7] which has been fully endorsed by EBJIS, MSIS and ESGIAI. We considered only patients who fulfilled the confirmed infection criteria.
2. *Infection control*, based on Delphi-method consensus criteria [8]: **a)** healed wound without fistula or drainage and no infection recurrence caused by the same organism strain; **b**) no subsequent surgical intervention for infection after reimplantation surgery, **c**) no PJI-related mortality. Necessity for suppressive antibiotic treatment or onset of another PJI caused by a different microorganism were also deemed treatment failures. Need for a non-infection-related early postoperative wound debridement (before discharge), due to haematoma, wound necrosis or wound dehiscence, was not considered a failure (regardless of culture results) if during follow-up there was no sign of infection recurrence.

#### Operative technique description

 Knee exchange arthroplasty was performed in a two-stage strategy by one of the four surgeons of our dedicated Infection Unit, each following the same surgical protocol.

**Fig. 1:**
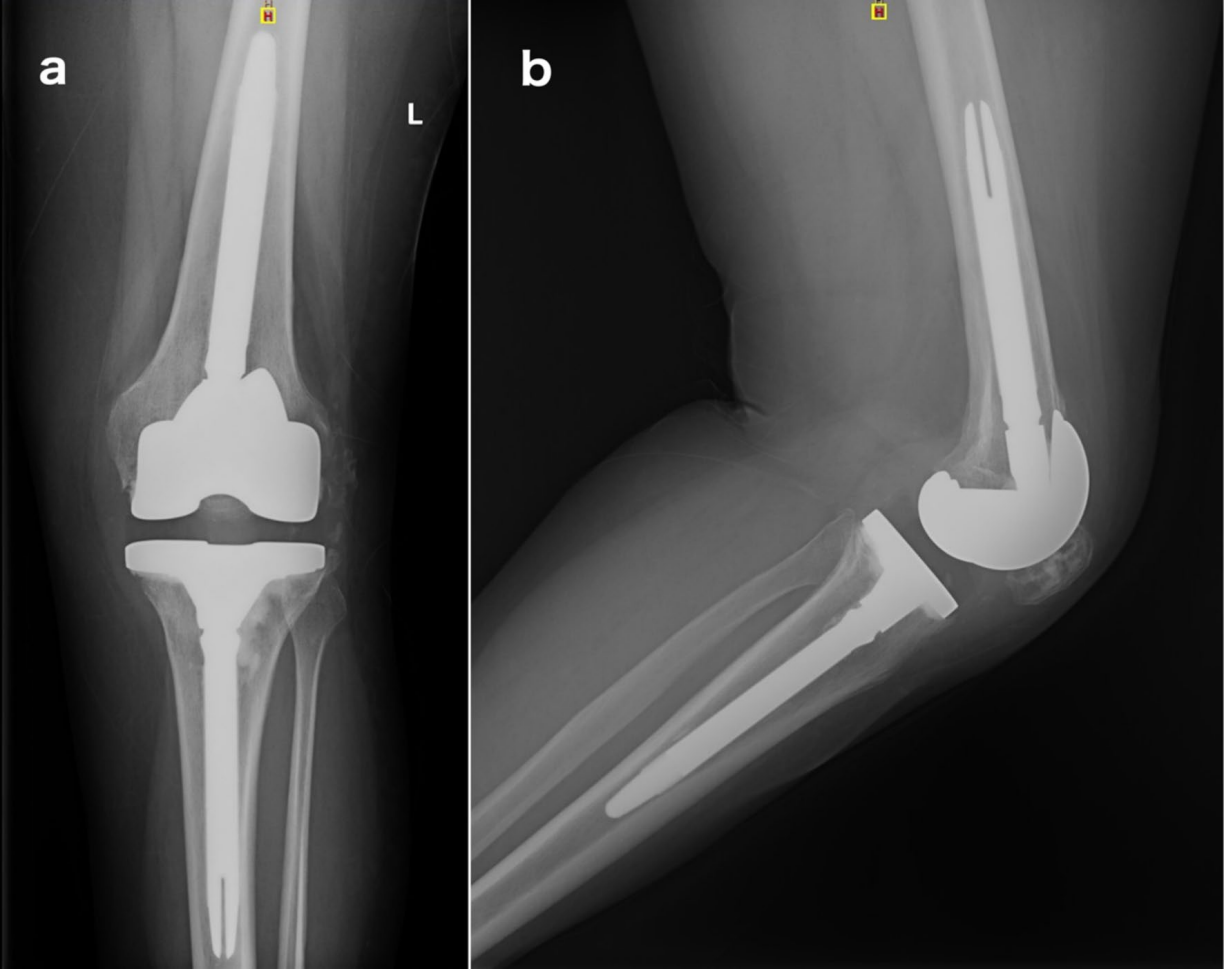
**a** Anteroposterior and **b** lateral pre-operative radiography of an infected (sinus tract) revision prosthesis due to Staphylococcus epidermidis

During the first stage a medial parapatellar approach was used and extended through a tibial tubercle osteotomy whenever a safe mobilization of the extensor mechanism could not be achieved or when correct visualization was compromised due to stiffness and rigidity [9]. The earlier prosthesis (Fig. 1) and cement were removed, and a thorough debridement was carried out.

**Fig. 2:**
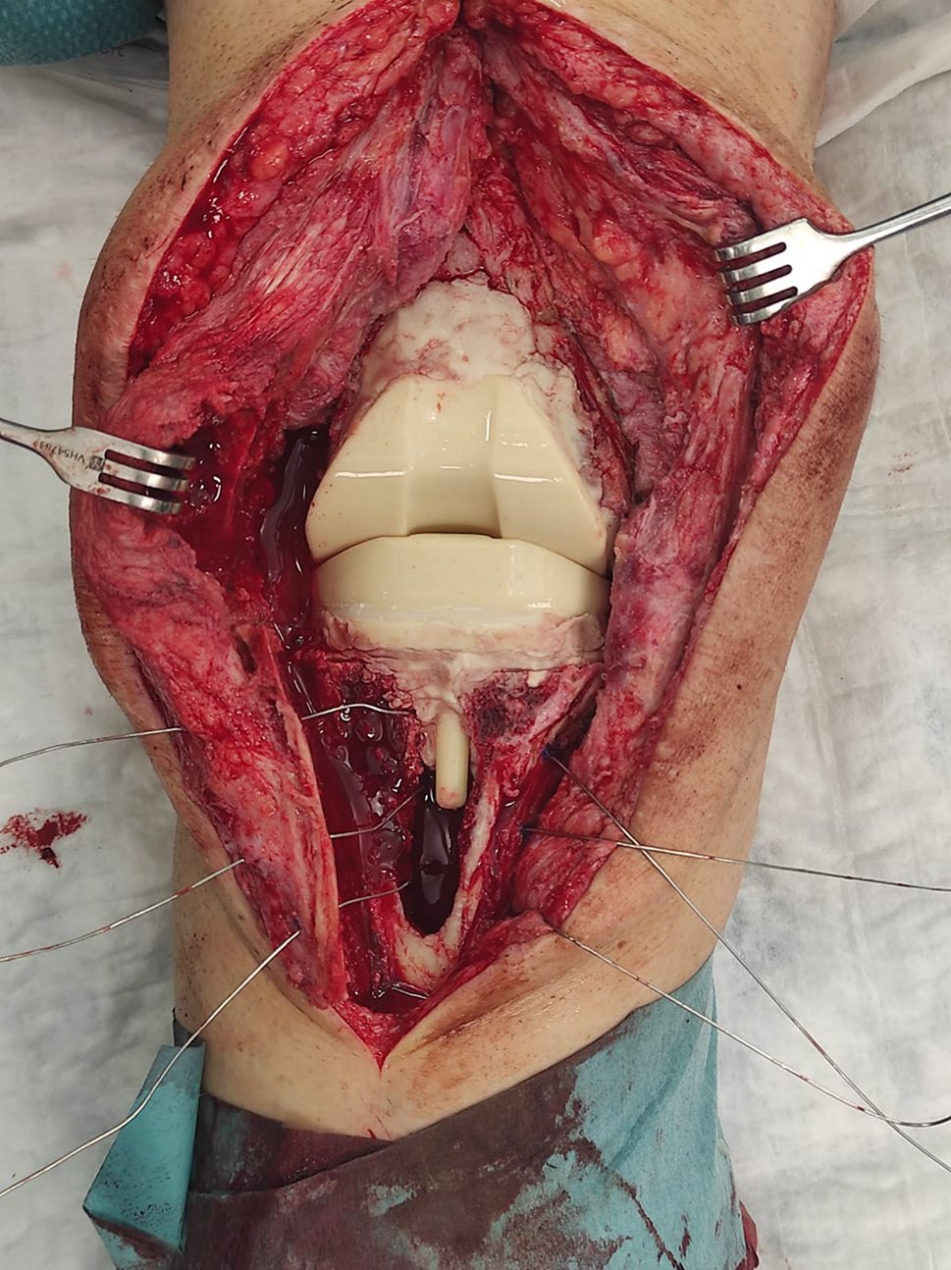
Vancomycin–gentamicin prefabricated antibiotic cement spacer (Vancogenx®, Tecres, Sommacampagna, Italy) fixed with a vancomycin-gentamicin-loaded acrylic bone cement (Vancogenx® bone cement, Tecres SpA, Sommacampagna, Verona, Italy)

**Fig. 3:**
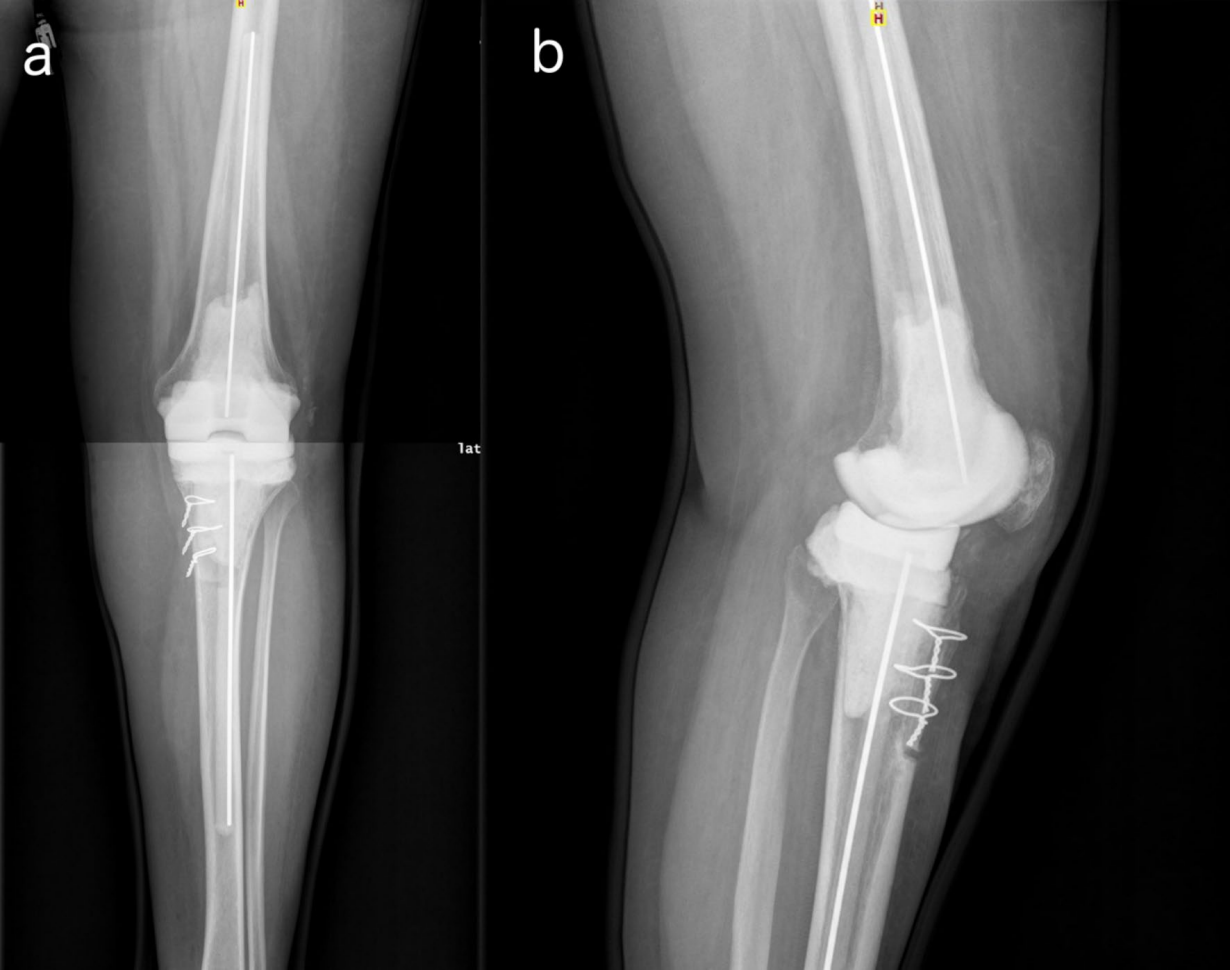
**a** Anteroposterior and **b** lateral radiography of a vancomycin–gentamicin prefabricated antibiotic cement spacer (Vancogenx®, Tecres, Sommacampagna, Italy). In some cases, aiming to increase stability and to fill the intramedullary dead space, a hand-made antibiotic-loaded cement stem (reinforced with a Steinmann pin) is connected to the spacers as can be seen in the radiographies

Before administering antibiotics, at least six specimens were taken for culture. All surgical fields were irrigated with saline without additives and using a low-pressure system, followed by implantation of a prefabricated antibiotic cement spacer (Tecres, Sommacampagna, Italy) (Figs. 2**, **3) fixed with a vancomycin-gentamicin-loaded acrylic bone cement (Vancogenx® bone cement, Tecres SpA, Sommacampagna, Verona, Italy) with an extra dose of antibiotic powder (specifically, 4 g of vancomycin and 4 g of tobramycin powder were added for each 40-g bag of standard-viscosity Vancogenx® bone cement). In cases of soft tissue deficiencies, extensor mechanism impairment or large bone defects, a hand-made static spacer was preferred [3].

All patients followed a post-operative antibiotic protocol guided by the infectious disease experts of our dedicated multidisciplinary unit. In general, the antibiotic treatment was selected following the clinical practice guidelines of the Spanish Society of Infectious Diseases and Clinical Microbiology (SEIMC), always considering the antibiogram of the bacteria present. Intravenous antibiotics were administered for 8–10 days. Usually a beta-lactam was used. If involvement of multi-resistant microorganism was suspected, a carbapenem, associated or not with a glycopeptide or a lipopeptide, was preferred. When final microbiological data was available and proper wound healing was achieved, antibiotics were switched to oral and maintained for at least 6 weeks. If possible, a combination of rifampicin with a second antibiotic was used for gram-positive infection. In the case of staphylococcal infection, the preferred combination was rifampicin plus levofloxacin. In gram-negative infections, whenever susceptible, oral ciprofloxacin was administered. According to our protocol, the second-stage procedure was done only when there was no clinical recurrence of the infection, the patient presented a nutritional recovery (optimization of pre-operative albumin and total protein blood levels, and optimal blood glucose control), and a decreasing trend in C-reactive protein was observed.

**Fig. 4:**
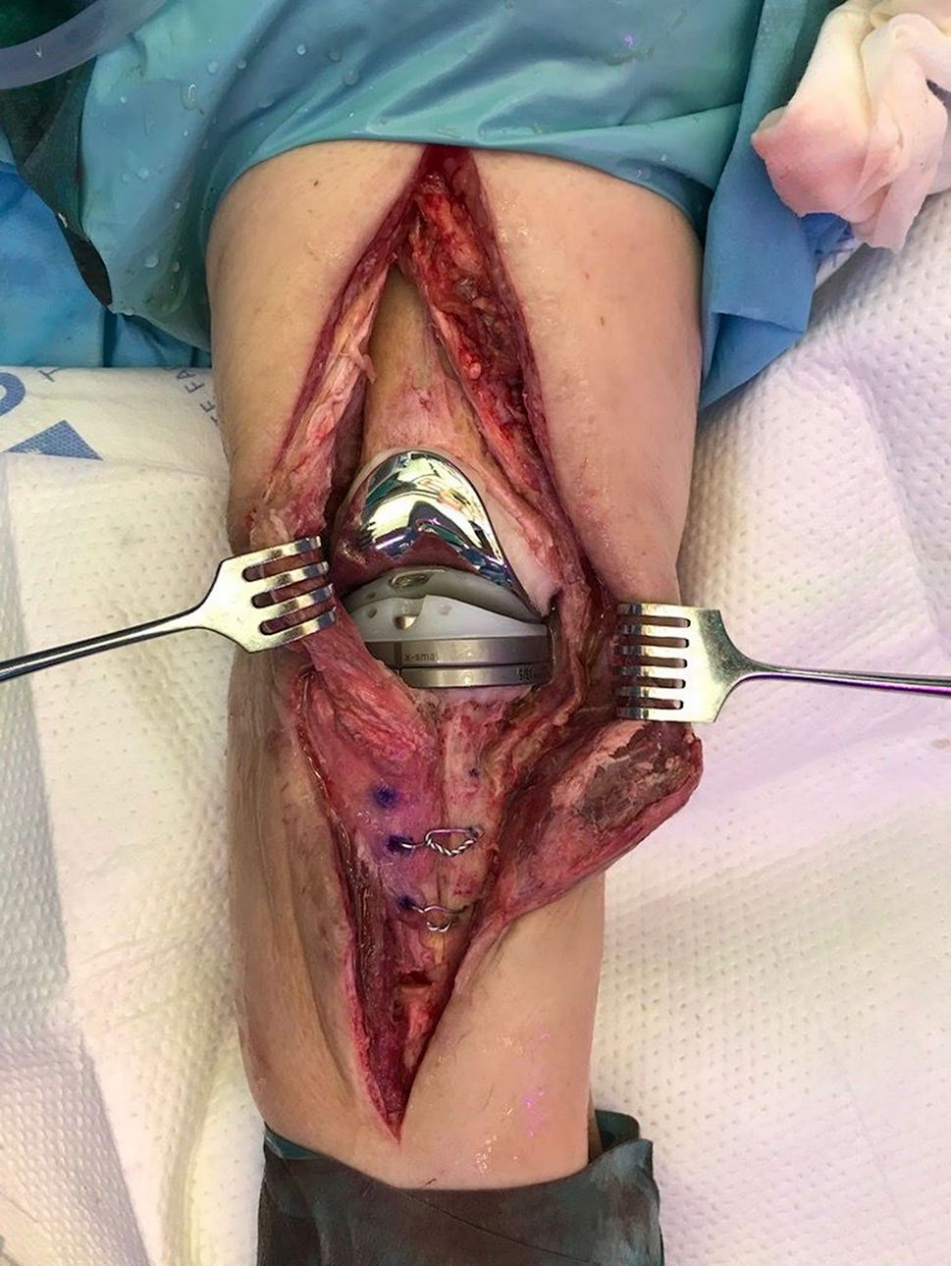
Cemented Endo-Model®-M knee implant (Waldemar Link GmbH & Co.®; Hamburg; Germany)

**Fig. 5:**
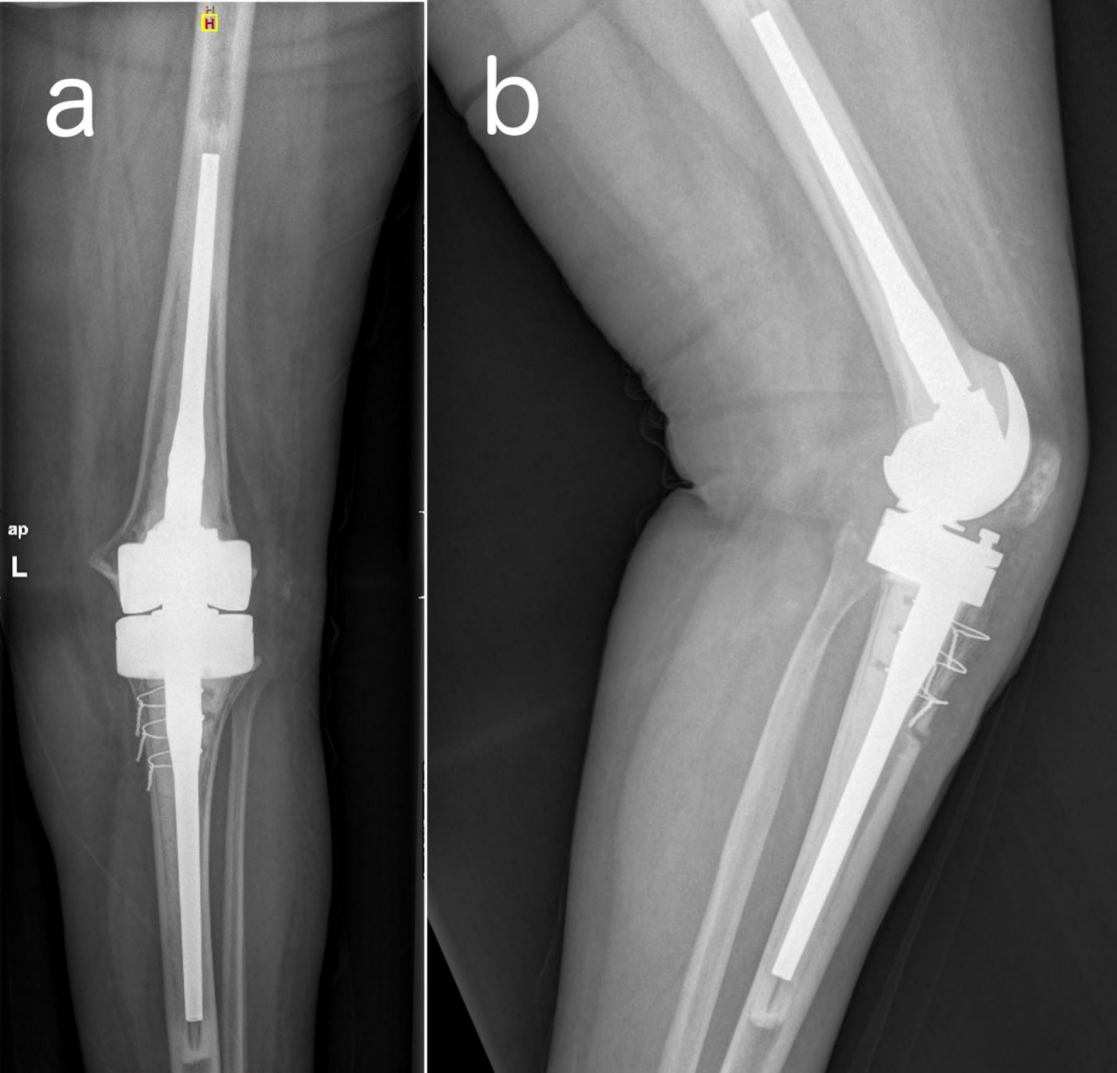
**a** Anteroposterior and **b** lateral post-operative radiography showing an Endo-Model®-M knee revision prosthesis

In the second stage, the spacer was removed, a second aggressive debridement was performed, and samples were collected. Joint reconstruction was done by implantation of a cemented Endo-Model®-M knee implant (Waldemar Link GmbH & Co.®; Hamburg; Germany) and fixed with a vancomycin-gentamicin preloaded prefabricated bone cement (Vancogenx® bone cement, Tecres SpA, Sommacampagna, Verona, Italy) without an extra dose of antibiotics. The articular components of the prosthesis are available in four sizes (x-small, small, medium and large) with the option of adding femoral and/or tibial titanium augments in cases with compromised bone stock. The cemented modular stems are available in lengths of 50–280 mm, and are joined to the articular component by a cone assembly (Figs. 4, 5). In no case tibial or femoral cones were used, as this type of metaphyseal supplementation technology was not available in our country during the study period.

**Fig. 6:**
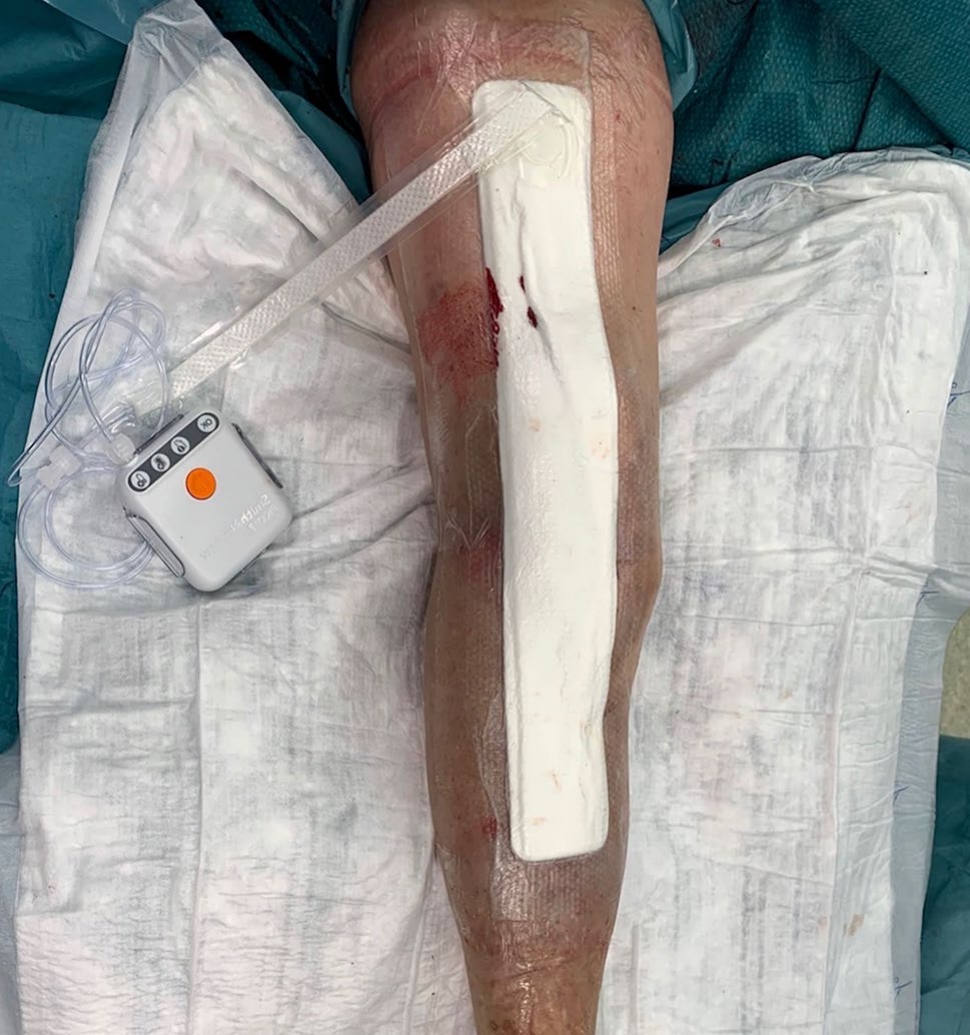
Prophylactic incisional Negative Pressure Wound Therapy -iNPWT (PICO®, Smith&Nephew; Memphis, USA)

After closure, prophylactic incisional Negative Pressure Wound Therapy (iNPWT) (PICO®, Smith & Nephew; Memphis, USA) is frequently used (especially if a comorbidity related to impaired wound healing is present or in patients with soft tissue deficiencies, as long as a flap surgery is not needed), for a minimum of 14 days (Fig. 6).

### Statistical analysis

Description of demographic and clinical characteristics was done using counts and percentages for categorical variables, and means and confidence intervals for continuous variables. Normality was tested using the Kolmogorov–Smirnov test. Groups were compared using the Chi-square test or Fisher exact test for categorical variables. The student t-test and ANOVA test, or the Wilcoxon–Mann–Whitney test and Kruskal–Wallis test, were carried out for continuous variables, depending on data distribution. All p values were two-tailed. *P*-values < 0.05 were considered statistically significant. Probability of implant survival was represented with a Kaplan–Meier graph. All the aforementioned tests were carried out using R software (R Development Core Team, version 4.1.3).

## Results

**Fig. 7:**
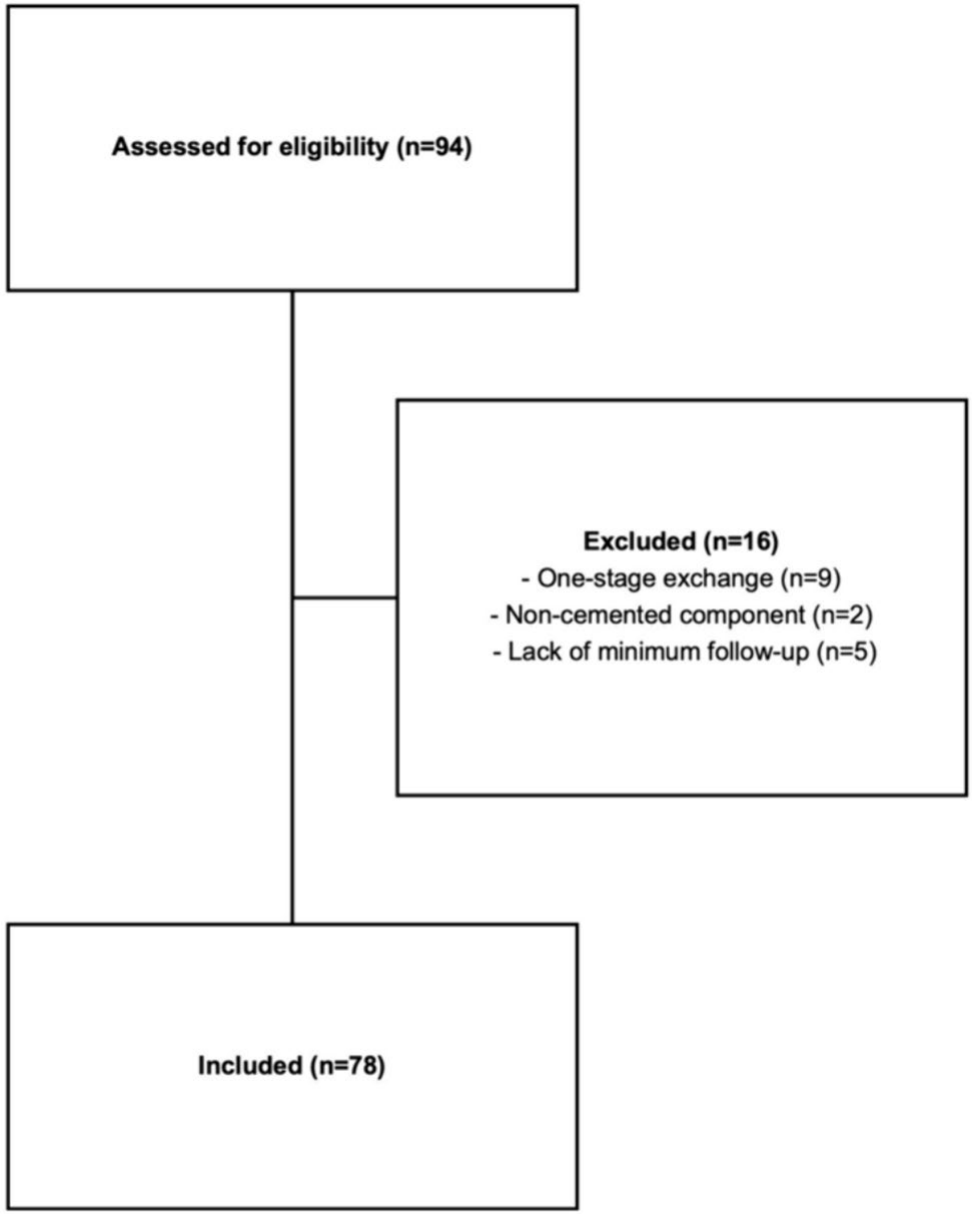
Flowchart of study cases

In our database review, 94 cases of knee PJI managed with an Endo-Model®-M implant were identified. After excluding ineligible cases, 78 patients were finally included (Fig. 7), with an average patient age of 70.5 ± 9.2 years. Comorbidities and previous surgeries were common among the study population. Patient characteristics are detailed in Table 1.

**Table 1:** Demographics, comorbidities and infection characteristics of the 78 patients studied

Variables	All patients N = 78 (100%)	Success N = 68 (87.2%)	Failure N = 10 (12.8%)	*p*
Age, years; mean (IQR range)	70.5 (46–86)	70.9	67.7	0.116
Sex, male/female	35(44.9) / 43(55.1)	30(44.1) / 38(55.9)	5(50) /5(50)	0.746
Diabetes mellitus	22(28.2)	19(27.9)	3(30)	1
Smoking	4(5.1)	4(5.9)	0	1
Obesity	18(23.1)	13(19.1)	5(50)	**0.045**
Alcoholism	2(2.6)	2(2.9)	0	1
Malignant neoplasm	11(14.1)	7(10.3)	4(40)	**0.03**
Arthritis	5(6.4)	5(7.4)	0	1
Cirrhosis	1(1.3)	1(1.5)	0	1
Coagulation problems	8(10.3)	6(8.8)	2(20)	0.53
Charlson Comorbidity Index				
≤ 2	20(25.6)	17(25.0)	3(30)	0.365
3–5	42(53.8)	38(55.9)	4(40)	
6–8	11(14.1)	8(11.8)	3(30)	
≥ 9	5(6.4)	5(7.4)	0	
ASA Scale				
I	2(2.6)	2(2.9)	0	
II	31(39.7)	27(39.7)	4(40)	0.476
III	43(55.1)	38(55.9)	5(50)	
IV	2(2.6)	1(1.5)	1(10)	
McPherson				
Type A	34(43.6)	30(44.1)	4(40)	0.678
Type B	38(48.7)	32(47.1)	6(60)	
Type C	6(7.7)	6(8.8)	0	
Infection scenario type				
Revision prosthetic infection	13(16.7)	11(16.2)	2(20)	0.091
Primary prosthetic infection	46(59.0)	41(60.3)	5(50)	
Primary prosthesis with previous failed DAIR	11(14.1)	11(16.2)	0	
Relapse of previous replacement due to infection	8(10.3)	5(7.4)	3(30)	
N surgeries previous to first stage; mean (IQR range)	2.06 (0–6)	2.0	2.7	0.104

Statistically significant *p* values are in bold (*p* < 0.05)

First-stage variables such as type of spacer used and PJI-causing microorganisms can be found in Table 2.

**Table 2:** Univariate analysis of variables investigated as predictors of failure in two-stage reimplantation

Variables	All patients N = 78 (100%)	Success N = 68 (87.2%)	Failure N = 10 (12.8%)	*p*
Spacer type				
Premade Vancomycin-Gentamicin with stem	34 (43.6)	30 (44.1)	4 (40)	0.663
Premade Vancomycin-Gentamicin without stem	24 (30.8)	20 (29.4)	4 (40)	
Premade Gentamicin	3 (3.8)	2 (2.9)	1 (10)	
Self-made static spacer	17 (21.8)	16 (23.5)	1 (10)	
PJI-causing microorganisms				
CoNS	23 (29.5)	17 (25)	6 (60)	0.434
*P. Acnes*	7 (9)	7 (10.3)	0	
Methicillin-sensitive *S. Aureus*	4 (5.1)	4 (5.9)	0	
*E. Faecalis*	2 (2.6)	1 (1.5)	1 (10)	
*Pseudomonas* spp.	2 (2.6)	2 (2.9)	0	
*Streptococcus* spp.	2 (2.6)	1 (1.5)	1 (10)	
Other microorganisms (single-isolated)	6 (7.7)	6 (8.8)	0	
Polymicrobial	11 (14.1)	11 (16.2)	0	
Negative cultures	21 (26.9)	19 (27.9)	2 (20)	
First stage approach				
EMP	35 (44.9)	30 (44.1)	5 (50)	0.746
TTO	43 (55.1)	38 (55.9)	5 (50)	
Second stage approach				
EMP	21 (26.9)	16 (23.5)	5 (50)	0.112
TTO	57 (73.1)	52 (76.5)	5 (50)	
Articular component size				
X-Small	6 (7.7)	5 (7.5)	1 (10)	0.294
Small	47 (60.3)	43 (64.2)	4 (40)	
Medium	24 (30.8)	19 (28.4)	5 (50)	
Femoral augments				
Yes	2 (2.6)	2 (2.9)	0	1
No	76 (97.4)	66 (97.1)	10 (100)	
Tibial augments				
Yes	45 (57.7)	37 (54.4)	8 (80)	0.177
No	33 (42.3)	31 (45.6)	2 (20)	
Wound dressing after second stage				
iNPWT	40 (51.3)	38 (55.9)	2 (20)	**0.042**
Standard wound dressing	38 (48.7)	30 (44.1)	8 (80)	
Complications after second stage				
Cutaneous necrosis				1
Yes	2 (2.6)	2 (2.9)	0	
No	76 (97.4)	66 (97.1)	10 (100)	
Need for early debridement				**0.03**
Yes	11 (14.1)	7 (10.3)	4 (40)	
No	67 (85.9)	61 (89.7)	6 (60)	

Statistically significant *p* values are in bold (*p* < 0.05)

*PJI* periprosthetic joint infection, *CoNS* coagulase-negative *Staphylococcus*, *EMP* extensile medial parapatellar, *TTO* tibial tubercle osteotomy, *iNPWT* incisional Negative Pressure Wound Therapy

Regarding the Endo-Model®-M components chosen during the index procedure, a small-sized articular component was used in 60.3% (47/78) of the cases, with an average femoral and tibial stem length of 147.7 ± 22.6 cm and 156.4 ± 20.7 cm, respectively. Prophylactic iNPWT (PICO®) was used in 51.3% (40/78) of the patients. Further information on second-stage variables can be found in Table 2.

After the second stage, complications included two cases of cutaneous necrosis (2.6%; 2/78), and need for early post-operative, non-infection-related, wound debridement in eleven cases (14.1%; 11/78), due to either haematoma (27.3%; 3/11), extensive wound necrosis (18.2%; 2/11) or wound dehiscence (54.5%; 6/11). Per protocol, during the debridement, cultures were taken, being unexpectedly positive in six cases (54.5%; 6/11); in all such cases isolated microorganisms differed from the ones found in the first stage. Need for an extra course of antibiotic was discussed with our infectious disease experts and was deemed necessary in five out of these six cases.

Regarding the principal end-point of our study, the overall infection control rate after our two-stage exchange protocol with a minimum follow-up of 24 months (range 24–136.5 months) was of 87.2% (68/78). On univariate analysis, obesity (p:0.045), history of cancer (p:0.03) and need for a debridement after the second stage (p:0.03) were identified as predictors of failure. Interestingly, use of iNPWT seems to have a positive effect against infection relapse (*p:* 0.042) (Table 3).

**Table 3:** Variables detected as predictors of success/failure in our two-stage reimplantation protocol on univariate analysis

Variables	All patients N = 78 (100%)	Success N = 68 (87.2%)	Failure N = 10 (12.8%)	*p*
Patient comorbidities				
Obesity (BMI ≥ 30)	18 (23.1)	13/18 (72.2%)	5/18 (27.8%)	**0.045**
Non-obese patients (BMI < 30)	60 (76.9)	55/60 (91.7%)	5/60 (8.3%)	
Malignant neoplasm history	11 (14.1)	7/11 (63.6%)	4/11 (36.4%)	**0.03**
Absence of malignant neoplasm history	67 (85.9)	61/67 (91%)	6/67 (9%)	
Protocol variables				
Use of iNPWT after second stage	40 (51.3)	38/40 (95%)	2/40 (5%)	**0.042**
Use of standard wound dressing after second stage	38 (48.7)	30/38 (78.9%)	8/38 (21.1%)	
Complications after second stage				
Need for surgical debridement	11 (14.1)	7/11 (63.6%)	4/11 (36.4%)	**0.03**
No need for surgical debridement	67 (85.9)	61/67 (91%)	6/67 (9%)	

Statistically significant *p* values are in bold (*p* < 0.05)

*BMI* body mass index, *iNPWT* incisional Negative Pressure Wound Therapy

**Fig. 8:**
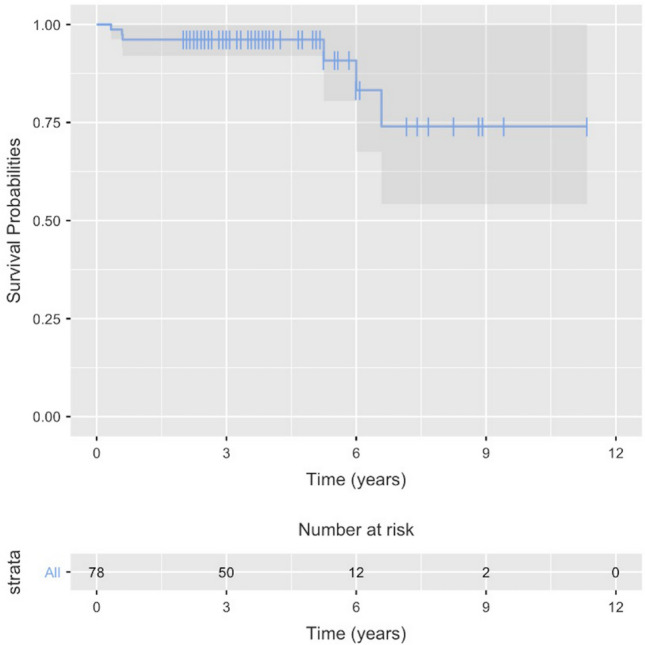
Kaplan–Meier curve. Probability of implant survival

Regarding implant survivorship, the Endo-Model®-M prosthesis was deemed to have failed if it was revised or removed, as occurred in six patients; overall implant survival was 92.3% (72/78). All failures were due to infection recurrence; no aseptic loosening was detected, nor were there any problems with the modular interface. Implant failure was highest during the first post-operative year, with three of the revisions occurring during this period. The other three implants left were removed more than 5 years after the second stage, the last one being revised at almost 7 years after reimplantation. This gives us a cumulative implant survival rate of 96.2% at 1 and 5 years, and 92.3% at final follow-up. Leaving septic loosening aside, implant survival was 100% at the completion of follow-up. In addition, a Kaplan Meier curve was built to assess probability of implant survival, showing this probability as 96.2% and 74.1% at 5 and 10 years, respectively, after reimplantation (Fig. 8).

## Discussion

As regards primary end-points, in this series of chronic knee PJI managed with a two-staged protocol with an Endo-Model®-M prosthesis as the final implant, we found an overall infection control rate of 87.2% (68/78), and an implant survival rate of 92.3% (72/78), with a mean follow-up of 4.3 years. The CMRH design demonstrated excellent short-to-medium-term survivorship (cumulative implant survival rate of 96.2% at 5 years) with no aseptic loosening or modular interface failure identified in the series. To the best of our knowledge this is the largest series reporting such outcomes with the CMRH design in an infected scenario.

As mentioned, thorough joint debridement is the key step in any treatment protocol. However, aggressive debridement can affect the integrity of the knee collateral ligaments, and so influence the type of revision implant chosen for the reconstruction surgery. This is even more evident in multi-operated stiff knees or in cases of failed surgical attempts to eradicate the infection. In such difficult-to-treat scenarios, a thorough, aggressive debridement will almost inevitably produce an impairment of collateral ligament function [6]. We consider that planned use of a rotating-hinge implant allows for much more radical debridement, without raising undue concern over the final stability of the knee. The rationale for using a modular implant rather than its monoblock counterpart is the flexibility the modular implant offers for resolving the frequently associated bone defects, and for adapting the stem length to the particular features of each case. We prefer the cemented instead of the non-cemented version due to the option of designing a tailored local antibiotic therapy using the antibiotic carrier features of antibiotic-loaded bone cement. The efficacy of such a protocol is supported by our results, with an overall infection control rate of 87.2% after a mean of 51.7 months in this difficult-to-treat infected scenario. Published results using the type of implant reported in this series for septic revision are scarce, and comparison with other, similar series is difficult, since, as far as we know, ours is the first published series on this modular implant design in cases of PJI. However, in the case of the non-modular Endomodel® implant, Pradhan et al. [10] studied 23 patients who had undergone a two-stage TKA exchange with implantation of a cemented rotational hinge prosthesis during the second stage. Of the 23 patients studied, 4 had an infection relapse. With a very similar philosophy to ours regarding aggressive debridement, but using a single-stage replacement strategy and a monoblock design, a series of 59 cases by Zahar et al. showed 93% infection control after a minimum follow-up of 9 years [6].

On univariate analysis, we detected some factors associated with increased risk of infection control failure (Table 3). Some are patient-related and thus difficult to modify, such as obesity (p:0.045) and history of cancer (p:0.03). The association between obesity and increased risk of PJI has been widely studied and seems consistent. Similar to our outcomes, results of a meta-analysis carried out by Guo et al. suggested that the risk of hip and knee two-stage exchange failure increased significantly in obese patients, with a higher association in studies performed in TKA [11]. Another study, designed as a two-to-one matched cohort study, specifically assessed the potential relationship between morbid obesity and increased risk of two-stage exchange failure, finding a 22% risk of reinfection in morbidly obese patients versus 4% risk in non-obese patients (p < 0.01) [12]. Regarding malignancy, there are several factors that increase the risk of infection in these patients, such as immunodeficiency, disruption of natural anatomic barriers, chemotherapy, radiotherapy and increased use of medical devices such as catheters [13]. However, few studies have found malignancy a significant risk factor for PJI or a predictor for bad outcomes after PJI treatment [14]. Although direct association between malignancy and increased failure risk after two-stage exchange is not clear, immunosuppression (a condition present in most cancer patients, albeit also in other pathologies such as inflammatory diseases or AIDS) has been found as a predictor of treatment failure after a two-stage exchange protocol in studies recently published [15, 16]. The need for any acute surgical wound debridement after the second stage (regardless of culture results) was found a predictor of failure in our study. Of the 11 patients who underwent this procedure, 63.6% (7/11) were infection-free at final follow-up, vs. 91% (61/67) when a debridement was not needed (p: 0.03). This fact would seem to be explained by the risk of surgical site contamination after any alteration of the skin barrier in the initial phase after surgery. A similar infection control rate (63.2%) was reported by Faschingbauer et al. [17] in their 19-case study of patients requiring debridement after a two-stage prosthesis replacement. However, as far as we know, there is no literature specifically assessing the relationship between infection control and the need for debridement after the second stage. On the contrary, the potential impact of the need for a debridement between stages has been studied by some authors, with controversial results [18, 19]. Further research is needed to clarify whether the need for debridement between stages or after the second stage constitutes a predictor of bad outcomes.

Interestingly, cases where prophylactic closed iNPWT was used during the second stage show better results in terms of infection control; p-value 0.042 (Table 3). Closed iNPWT has been associated with reduced wound complications after revision total joint arthroplasty [20, 21] but, to our knowledge, ours is the first investigation showing a significant improvement of knee PJI infection control following iNPWT use. Among the suggested advantages of iNPWT in the infected setting are mechanical stabilization of the wound (reduction of wound tension), decreased oedema, seroma and subcutaneous dead space, and increased blood flow and angiogenesis—undoubtedly favourable features in such a difficult-to-heal scenario. Based on our results, with all precautions and with the need for prospective randomized studies, we believe that the use of iNPWT in these complex cases is highly recommended; it has become the standard of care in our dedicated septic unit.

Currently available rotating-hinge devices yield substantially better clinical results and implant survivorship when compared to the suboptimal outcomes with earlier designs [10]. The survival rate of the Endo-Model®-M cemented knee prosthesis in our series was 92.3% (72/78), with a mean follow-up of 4.3 years and with only three revisions in the first year and another three at more than 5 years following two-stage exchange. Each of these instances was due to septic failure. The implant under study here has demonstrated excellent short-to-medium-term survivorship (cumulative implant survival rate of 96.2% at 1 and 5 years) with no major complications registered, and no instance of aseptic loosening or modular interface failure. Although the survivability of the Endo-Model® has been studied by several authors, the majority present results with non-modular implants. Sanguineti et al. [4] included both primary surgery (25 patients) and revision cases (20 patients), only five due to chronic PJI, finding a mean prosthesis survival of 95.5% and 93.3% at 1 and 5 years post-surgery, respectively. Brown et al. [5] included 41 primary surgery cases, 47 aseptic revisions and 12 two-stage septic revisions in their 100-patient series. They found a cumulative implant survival rate of 99% at 1 year and 95% at 5 years, with the type of arthroplasty (primary or revision) showing no significant effect on implant survival. Koch et al. [22], with a mean follow-up period of 58 months, reported a revision rate of 29.6% in their 109-case series, which included both monoblock and modular implants, with a revision rate due to infection recurrence of 20.6%; aseptic loosening was the cause of revision in 5.6% of their patients.

We consider that modularity offers an inherent advantage in this type of case, allowing for adaptation of the implant to each case’s particular circumstances. As with any other modular implant, concerns exist over the possibility of modular component failure. In our series of 78 modular cases, we have not observed a single case of failure in modular components of this particular design, supporting the safety of such an approach in chronically infected knee prosthesis cases. To the best of our knowledge, this feature has not been previously published.

We acknowledge the limitations of our study. The first lies in the study’s retrospective nature. Retrospective studies rely on chart notes from which important data may be lacking, potentially increasing bias incidence. A second major limitation is our lack of a comparison group; absence of a control group makes it impossible to compare results directly with those treated with another hinge design or non-hinged prosthesis. However, a comparative study is far removed from the objectives of the present study, which focuses on clinical outcomes of a specific comprehensive exchange arthroplasty protocol. Third, four different surgeons carried out the study’s procedures, inevitably adding some variability of results. To balance this fact to some degree, all four surgeons were familiar with the protocol described and followed it faithfully; additionally, regarding infection control rate and implant survivorship, no statistically significant differences were found between them. Other limitations to consider are the sample size and the follow-up period; even so, both of these factors were comparable or superior to those of previously published studies. Although the limited sample size impeded a solid multivariable regression analysis, to our knowledge this is the largest series assessing the results of the Endo-Model® M prosthesis when used as the final implant in a two-stage exchange protocol. Finally, we recognize that all our patients were treated at a single, high-volume, specialized centre. It is difficult to extrapolate our results to smaller or less-experienced units. Ideally, patients such as these should be referred only to centres with extensive experience in treating this type of complex case.

## Conclusions

In conclusion, our results suggest that the use of a CMRH design in a two-stage exchange arthroplasty strategy may be associated with high rates of infection control and an anecdotal incidence of aseptic loosening or modular component failure.

## Data Availability

The data that support the findings of this study are available from the corresponding author, upon reasonable request.
